# Generating synthetic multidimensional molecular time series data for machine learning: considerations

**DOI:** 10.3389/fsysb.2023.1188009

**Published:** 2023-07-25

**Authors:** Gary An, Chase Cockrell

**Affiliations:** Department of Surgery, University of Vermont Larner College of Medicine, Burlington, VT, United States

**Keywords:** synthetic data, time series data, artificial intelligence, artificial neural network, mechanistic modeling, agent-based model, machine learning, multiscale modeling

## Abstract

The use of synthetic data is recognized as a crucial step in the development of neural network-based Artificial Intelligence (AI) systems. While the methods for generating synthetic data for AI applications in other domains have a role in certain biomedical AI systems, primarily related to image processing, there is a critical gap in the generation of time series data for AI tasks where it is necessary to know how the system works. This is most pronounced in the ability to generate synthetic multi-dimensional molecular time series data (subsequently referred to as synthetic mediator trajectories or SMTs); this is the type of data that underpins research into biomarkers and mediator signatures for forecasting various diseases and is an essential component of the drug development pipeline. We argue the insufficiency of statistical and data-centric machine learning (ML) means of generating this type of synthetic data is due to a combination of factors: perpetual data sparsity due to the Curse of Dimensionality, the inapplicability of the Central Limit Theorem in terms of making assumptions about the statistical distributions of this type of data, and the inability to use *ab initio* simulations due to the state of perpetual epistemic incompleteness in cellular/molecular biology. Alternatively, we present a rationale for using complex multi-scale mechanism-based simulation models, constructed and operated on to account for perpetual epistemic incompleteness and the need to provide maximal expansiveness in concordance with the Maximal Entropy Principle. These procedures provide for the generation of SMT that minimizes the known shortcomings associated with neural network AI systems, namely overfitting and lack of generalizability. The generation of synthetic data that accounts for the identified factors of multi-dimensional time series data is an essential capability for the development of mediator-biomarker based AI forecasting systems, and therapeutic control development and optimization.

## 1 Introduction: what this article is, and is not, about

Synthetic data is recognized to be needed for machine learning (ML) and artificial intelligence (AI) systems to reach their full potential (Nikolenko, 2019). This article presents a hypothesis and theoretical basis regarding generating synthetic multi-dimensional molecular time series data (henceforth termed synthetic mediator trajectories or SMTs) for biomedical applications where it is critical *to have an understanding of how the system works*. Therefore:

• This article is about how to generate synthetic data to augment multiplexed molecular-level time series data used to forecast the behavioral trajectory of an individual patient or, perhaps more importantly, for the evaluation of whether novel drugs or novel combinations of existing drugs (repurposing) will work (hypothesis testing).
• This article is not about searching for a particular candidate compound based on a presumed target pathway/gene/molecule (hypothesis generation).
• This article concerns forecasting or evaluative tasks that presume a mechanistic, hierarchical causal relationship between the lower scale features (cellular/molecular) and the higher order, system-level phenotype (clinical disease manifestation) (Bareinboim, 2020).
• This article is not about producing and using structural causal models whose feature sets are scale-agnostic and flat (as is seen in utilizing electronic healthcare data that concatenates demographic, physiologic and laboratory data to produce synthetic populations) (Pearl, 2010).
• The task of generating SMTs focuses on enhancing the output of experimental cellular-molecular biology, which reflects the vast majority of biomedical research on the discovery and development of potential drugs.
• This article is not about using physics-based equations to simulate biology in systems that can be represented at a mechanical-physical level, such as fluid dynamics models of vascular flow or the material-wear properties of mechanical joints.
• This article is about generating synthetic data that can allow for the identification of individual trajectories (e.g., personalization).
• This article is not about synthesizing aggregate populations (“virtual populations”), as would be seen in epidemiological models.

We hypothesize that generating multi-scale, hierarchical time series data precludes the use of: 1) statistical methods, including the use of generative adversarial neural networks (GANs) and 2) existing approaches that generate virtual/synthetic populations that, while producing heterogeneous synthetic populations, do so by aggregating classes of patients around mean mediator values and do not replicate the individual patient trajectories needed for ML/AI training (Brown et al, 2015; Renardy and Kirschner, 2020; Jenner et al, 2021; Sips et al, 2022). We further hypothesize that these tasks require the use of sufficiently complex, mechanism-based simulation models of cellular and molecular processes to generate SMTs to train artificial neural networks (ANNs)/AI systems to address these tasks. As this is a Hypothesis and Theory article, the arguments presented involve:

1. Challenging underlying assumptions regarding what statistical approaches can be used (specifically the assumption of normal distributions predicated upon the applicability of the Central Limit Theorem.
2. Identifying known failure modes for ML/AI systems (specifically regarding the failure to generalize)
3. Presenting a proposed solution that addresses these known limitations.

We then present an example of how a sufficiently complex, mechanism-based simulation model can be used to generate SMT, but also acknowledge that at this point its benefits remain hypothetical, though grounded in theoretical principles that suggest its utility. To aid in navigating this paper, we provide the following outline of the components:

• Introduction to Synthetic Data in the wider, general community
  o How such Synthetic Data is generated
• Potential Applications of Synthetic Data in Biomedicine
  o Circumstances where general approaches are applicable
  o Circumstances where general approaches are not applicable, specifically with regards to multiplexed molecular time series data, and why
    ⁃ Why statistical approaches are insufficient
    ⁃ Why physics-based simulation is insufficient
• What is required for generating synthetic multiplexed molecular time series/molecular trajectories for the purposes of ML/AI training
  o Identifying the limitations of ML/AIs
  o Strategies to overcome the limitations of ML/AIs
• A proposed strategy for generating SMT, with an example
• Future Considerations

## 2 Synthetic data: general concepts, current uses and means of generation

First, we provide an overview of synthetic data, and use cases where its generation and use are on sound theoretical footing. Training ANNs for AI applications is notoriously data hungry, and even in areas with copious data there are recognized benefits to the supplementation of that data with synthetically generated data (Nikolenko, 2019). The most well-publicized examples of AI use synthetic data: image recognition/generation (Kortylewski, 2018), natural language processing/generation (Puri, 2020), self-driving cars (Bhandari, 2018) and game-playing systems (Silver et al, 2017; Vinyals et al, 2019; Perolat et al, 2022). In fact, the development of AI image generators and chatbots integrates the generation of synthetic data with the target applications, which are essentially means of generating “realistic” synthetic objects. The means of generating synthetic data falls into two general groups:

1. *Statistical* synthetic data can be generated if there is enough existing real-world data such that either: 1) the statistical distribution of system features can be reliably approximated or 2) an ANN can be trained, based on their property as Universal Approximators (Hornik et al, 1989), to a sufficiently robust generative function, an process that often utilizes generative adversarial neural networks or GANs (Bowles, 2018; Creswell et al, 2018). However, if an ANN is going to be used to approximate the generative function underlying the data, there needs to be sufficient existing training data such that a GAN-adversarial training scheme can distinguish between applied noise and the “true” data/invariant component (as determined by appropriate annotation). Consequently, this approach has found its greatest success in image analysis, where vast libraries of annotated images have been able to be used to both train initial ANNs and serve as reference points for GAN-driven synthetic image generation. The success in this field is evident in the two most successful applications generating synthetic images: Dall-E2 https://openai.com/product/dall-e-2 and Midjourney https://www.midjourney.com/home/).
2. *Simulation* generated synthetic data from mechanism-based simulation models can be considered “real enough” if the generative simulations are firmly grounded in natural laws (“physics-based”) or within the context of known rules (e.g., games). In these cases, there is a high degree of confidence in the rules and mechanisms of the simulations, and thus high trust in the fidelity of the synthetically generated data and the “real-world” in which the trained systems must operate (acknowledging that in the case of a game, the game itself represent the “real world” for the player).

These two successful forms of generating synthetic data are presented as reference points for our subsequent discussion to identify biomedical tasks for which they are suited, and those for which they are not.

## 3 Biomedical synthetic data: cases where existing methods can, and cannot, be used

There are biomedical use cases where the above well-accepted means of generating synthetic data through statistical or physics-based methods can be applied (Chen et al, 2021). Biomedical image processing (for either radiology or pathology) readily falls into the category of general image processing, and the same approaches used for image analysis can be extrapolated to biomedical applications (Candemir et al, 2021; Chen et al, 2021; Kitamura et al, 2021; Kelly et al, 2022; McAlpine et al, 2022; Seah et al, 2022; Daniel, 2023). Other circumstances where there may be enough existing data for statistical distributions are population-level data suitable to represent the control population in a potential clinical trial (Zand et al, 2018; Galaznik et al, 2019; Myles et al, 2023) or based on data from electronic health records (Baowaly et al, 2019; Chin-Cheong et al, 2019; Tucker et al, 2020; Libbi et al, 2021; Hernandez et al, 2022; Venugopal et al, 2022). In terms of simulated synthetic data, biomedical systems that can be represented as physical systems, such as fluid dynamics for anatomic representation of blood flow, electrical circuits for cardiac conduction, or the mechanical properties of joints, can be simulated with well-recognized “physics-based” methods (Levine, 2019; Burton et al, 2021; Peng et al, 2021; Sharma et al, 2022).

However, for the vast majority of biomedical research, namely experimental cellular and molecular biology, neither of these conditions hold. A central premise of experimental biomedical research is that more granular mechanistic knowledge can lead to improved human health, i.e., through the development and use of various-omics-based and multiplexed molecular assays. The foundation of the drug development endeavor is based on the premise that more detailed molecular knowledge of biological processes is the means to identifying more effective and precise new therapeutic agents. However, this experimental paradigm has two consequences that challenge the application of statistical, data-centric forms of analysis and, consequently, the ability to generate synthetic data.

1. Perpetual Data Sparsity. As each new “feature” (gene, biomarker, etc.) is identified, this adds dimensionality to the characterization of biological systems and this process carries with it a cost: the Curse of Dimensionality (Verleysen and François, 2005). The Curse of Dimensionality means that with each additional feature used to describe a system, there is an exponential increase in the potential configurations those features can take relative to each other (combinatorial explosion) and, similarly, the amount of data/sample points needed to characterize those potential combinations; *this leads to a state of perpetual data sparsity.* Because the space of potential combinations is perpetually under-sampled in the real world, it is not possible to generate the “true” statistical distribution of these values. This is specifically relevant to multiplexed molecular measurements, which are used because there is not a single measured entity with enough discriminatory power/utility (or else the multiplexed or-omics characterization would not be needed).
2. The inapplicability of the (Central Limit Theorem, 2008). Often there is an assumption that biological data will follow a normal distribution. This assumption is based on the presumption that the data being collected is governed by the Central Limit Theorem. However, the fundamental requirement of the application of the Central Limit Theorem is that the measurements are *independent random* variables. For molecular measurements related to disease this is not the case for the following reasons: 1) that the measured entities are not independent of each other is readily evident insomuch these molecules/mediators/genes are almost always connected by shared pathways, so the value of one entity will affect the value of another, and 2) the source of the samples are not random, given that the sampling occurs in a population that is preselected based on their manifestation of a disease process. Given that the initial requirements for application of the Central Limit Theorem, which is the justification for assuming a normal/Gaussian distribution of values, are not met, this means that one cannot assume a normal distribution for this type of data. This finding has been confirmed in several publications (Hardin and Wilson, 2009; Posekany et al, 2011; Lubura et al, 2012). Note that this does not mean that such measurements will not be normally distributed, but that such a normal distribution cannot be assumed.

Because statistical/data-centric approaches have the above fundamental limitations in terms of generating SMT, we assert that mechanism-based simulation methods need to be applied. However, there are also specific issues to directly translating the concept of “physics-based” simulations to those that can be used to represent the multiscale effects of cellular/molecular biology.

1. Multiscale cellular/molecular biology simulations cannot be produced *ab initio.* Biology, as a physical system, is certainly bound by the fundamental laws of physics and chemistry, but there are no corresponding fundamental laws that constrain the dynamics and output of cellular/molecular biology. While certain physical laws and constraints can be incorporated into cellular/molecular simulations (i.e., mechanical effects on cell signaling or mass conservation in metabolism), these components must inevitably be connected to representations of how cells respond to and modulate their behavior to these features. It is these behavioral aspects of cellular/molecular biology that produce the richness of biology, and for which there are not constraining fundamental laws. Because of this, the essential features of cellular/molecular biology cannot be represented by “physics-based” simulations (Levine, 2019; Burton et al, 2021; Peng et al, 2021; Sharma et al, 2022). Ultimately, the fundamental problem is that the generative mechanisms that lead to molecular mediator time series data are mostly unknown; further, they are undiscoverable (at present) by theory because the set of events that generate molecular mediator time series data are not tractable to compute *ab initio* (i.e., from the electronic configurations of the relevant molecules).
2. Dealing with the impact of perpetual epistemic uncertainty and incompleteness. Because of the lack of cell-behavior-scale fundamental laws, there is perpetual epistemic uncertainty in terms of the molecular-cellular rules that govern the biological system. Pushing the boundary of knowledge is the entire goal of cellular/molecular biology, but it must be acknowledged that it is impossible to know everything. Therefore, in terms of applied biology there must be some way of using incomplete knowledge to in a useful fashion. The only means of accomplishing this is by posing a particular mechanistic hypothesis and then operating on that hypothesis structure (through iterative experiment, recalibration/validation and iterative refinement) until it is proven to be insufficient. Establishing generative hierarchically causal/multi-scale mechanisms for such simulation models involves 1) identifying a level of abstraction that is “sufficiently complex”, and 2) utilizing a simulation model use strategy that provides the least bias, and therefore greatest explanatory expansiveness, in terms of determining its link to the real-world. This latter concept is reflected in the Maximum Entropy Principle, a formal method that is grounded in Information Theory and Statistical Physics (De Martino and De Martino, 2018), which utilizes thermodynamic principles to describe why any computational model of a biological system must necessarily have multiple parameterizations. An expansion of the Maximal Entropy Principle is manifested in our calibration methods (see below in Section 4) aimed at finding simulation model parameterizations and configurations that cannot be falsified by existing data.

Thus, the investigatory paradigm in experimental biology generates conditions that preclude the application of traditional means of generating synthetic data to producing SMTs:

1. Sparsity of high-dimensional time series data renders any assumptions regarding the true statistical distribution problematic.
2. Simulation models representing the SMT cannot be generated from physics-based fundamental laws.
3. The generative rule structures of potential mechanism-based simulations have perpetual epistemic uncertainty regarding biological rules.

Because of these factors, methods for generating SMT using either statistical methods or physics-based/ground-truth/*ab initio* simulations cannot be used. Therefore, an alternative approach is necessary, one that accounts for the limits of ANN-based AI systems and the perpetual epistemic uncertainty/incompleteness regarding cellular and molecular mechanisms.

## 4 What features must synthetic mediator trajectories (SMTs) have?

We identify two main classes of issues to address in generating SMT for the purpose of ML training. The first is mitigating the failure modes for ANN AI systems; these establish issues that need to be overcome by a simulation strategy to generate SMT:

1. ANNs fail to generalize. This is due to ANN training data sets not being sufficiently and comprehensively representative enough of the possible data configurations in the real world. In these cases, the discrepancy between the training set and the eventual application in the real world leads to the condition termed data drift (Nelson, 2015; Baier et al., 2019; Ackerman et al, 2020). Overcoming this limitation arising from insufficient training data is exactly the goal of using synthetic data for ML/AI, but with the recognition that such synthetic data must be generated in a fashion that provides a more comprehensive, expansive representation of the real-world data, i.e., data augmentation (which is the point of this paper). Therefore, the means of generating SMT should be as expansive (e.g., *least biased*) as possible, representing as much of the breadth of possible configurations of the target/real-world system, so the generative function learned by the ANN is applicable to the widest possible circumstances that might exist in the real world (thereby mitigating and forestalling data drift and increasing the robustness and generalizability of the trained ANN).
2. Inability to discriminate. A significant number of AI tasks involve distinguishing between one group from another. As such, there is an initial supposition that there is a detectable difference present between groups via a distinguishable phenotype/outcome. The issue with much existing time series data is that the range of values within each group is nearly always larger that the difference between some statistically determined characteristic of each group (be it mean or median). Since that the “true” statistical distributions are not known, the only way to create synthetic data that represents each different group is to generate it mechanistically, given the criteria of non-falsifiability by existing data and expansiveness in terms of potential explanations given a particular hypothesis structure (e.g., dealing with epistemic uncertainty as per the Maximal Entropy Principle).

Overcoming these two limitations intrinsic to ANNs are therefore key goals for the construction and use of a putative mechanism-based multiscale simulation approach to generating SMT to be used in ML training. Combining these ANN-limit mitigation requirements with the need to deal with perpetual epistemic uncertainty in simulation model structure leads to the following proposed requirements, with accompanying rationale, below:

1. Choosing a “sufficiently complex” abstraction level for the multiscale mechanism-based simulation model. All computational models incorporate parameters within the rules/equations that make up the model. In mechanism-based multi-scale simulation models of biological processes those rules often represent cellular functions and molecular events, such as receptor binding, signaling, gene activation, protein synthesis or secretion, etc. However, these models do not explicitly represent every component of every step present in the cell; in practice this is impossible because the sum-total of interactions between components, or even the total set of components, is not known. However, it is possible to construct such models where the unknown features (equivalent to latent variables) can be structured such that these features represent (primarily) the *responsiveness* of the represented functions in the model. We therefore consider a “sufficiently” complex multi-scale mechanism-based model as one where all essential cellular behavioral functions for a given purpose of the models are represented via a selection of chosen pathways, but with a latent space of variables that represent the differential responsiveness/gain of the represented functions (representing in aggregate unknown/unidentified genetic, epigenetic or signaling pathways not explicitly represented in the model). If such a model structure is followed, then the unknown features/latent variables can be aggregated into a multi-dimensional set of configurations that are responsible for generating the data heterogeneity seen across biological populations (Cockrell and An, 2021). Therefore, the next step is developing a means of operating on this model in a way that maximizes the expansiveness of behavioral representation used to generate SMT.
2. Maximizing the expansiveness of the generated SMT to minimize data drift for the ANN. It is readily evident that there can be no preconceived means of restricting the possible configurations of these latent responsiveness features. What is needed is a means of minimizing potential *a priori* bias in the representation of this latent space. The concept of minimizing bias by maximally quantifying ignorance is the fundamental basis for the Maximum Entropy Principle. While our proposed approach does not directly and formally apply the Maximum Entropy Principle to synthetic data generation, the general principle of not restricting the range of possible system configurations by some means outside the available data is crucial to mitigate overfitting during training of the ANN. A key difference in our proposed approach is that we operate from the starting point of a knowledge-based hypothesis structure that is embodied by the simulation model and utilize the latent space of unrepresented features, represented in a mathematical object, as the means of maximizing ignorance/entropy (in an information theoretic sense).
3. Translating expansiveness of representation (as per the Maximal Entropy Principle) into an alternative view of “calibration.” In general practice, the process of calibrating a computational model involves finding the minimally sufficient set of parameterizations able to “best” replicate an existing data set. Because the space of parameterizations is functionally infinite, there is an intrinsically reductive nature to this task; this reductive paradigm is also manifest in common practice of fitting to the mean of what is in reality a highly variable data set (Brown et al, 2015; Renardy and Kirschner, 2020; Jenner et al, 2021; Sips et al, 2022). If the goal of the modeling exercise is to provide some insight into the differences between different cohorts manifesting different phenotypes of interest (e.g., disease versus not disease), then this aggregating process can be useful and beneficial. However, if the goal is to then use that simulation model to produce synthetic data that captures the total amount of expressiveness in that data set (e.g., the heterogeneity of different trajectories manifesting as the variability seen in the data), then the aggregating/mean-fitting approach is insufficient. Rather, the “noisiness” of the data is exactly what must be encompassed by any calibration process used to describe the latent space of unrepresented features in a fashion that maximizes information-entropy. Additionally, the outlier values in a biological data set cannot be considered the min-max values for that particular variable/feature/molecule. Given the perpetual sparsity of these data sets, there can be no supposition that one may have happened to stumble across the actual maximal value biologically possible for that condition. Rather, the “outliers” in a data set need to be considered as only points within a larger potential distribution able to be generated by a proposed simulation model. Thus, any means of generating SMT must be able to expand the range of possible values for any feature in a biologically plausible fashion.

Therefore, we hypothesize that the generation of appropriate SMT for training an AI system requires a multi-scale mechanism-based simulation model that embodies an initial set of generative components and a means of operating over that knowledge structure that minimizes the bias regarding all the interactions (the latent variable space and interactions, including second, third to *n*-order effects) (De Martino and De Martino, 2018). The following section presents a demonstration of such an approach, which uses an agent-based model (ABM) constructed in a particular fashion, and a non-traditional conception of model “parameter space” that represents an initial approximation of a means of characterizing the latent space of unrepresented interactions for a given model. Note that this method of using ABMs, which addresses the generative hierarchical nature of SMT, is distinct from those approaches that use ABMs to generate virtual populations for epidemiological studies, where the trajectories of internal states of the agents is not the primary focus (Popper et al, 2020; Bissett et al, 2021; Truszkowska et al, 2021).

## 5 A proposed approach for generating SMTs

Here we present an example of a strategy for generating SMT to potentially train an AI for tasks that require either the forecasting of individual patient trajectories or the discovery and testing of potential interventions on molecular targets (e.g., novel drugs). This example is not intended to imply that the specific steps and methods listed below are the *only* means of meeting the above stated criteria for generating SMT, but does illustrate an approach that addresses all the required theoretical criteria for such synthetic data, as listed in the previous section:

1. The use of a mechanism-based multiscale simulation model grounded in existing biological knowledge. The use of a simulation model provides a means of partially addressing the Curse of Dimensionality, where the constraints on behavior enforced by the incorporated mechanistic rules constrain the multi-dimensional configurations possible. The multiscale nature of the simulation model overcomes the limits imposed by the Causal Hierarchy Theorem in terms of representing hierarchical generative causal relationships in a testable fashion.
2. The incorporation of epistemic uncertainty into the simulation model while following the Maximal Entropy Principle through the use of a mathematical object, the Model Rule Matrix, described in detail below.
3. Utilizing the concept of non-falsifiability in the generation of an unbiased, expansive synthetic data set, also as per the Maximal Entropy Principle, to overcome the inherent limitations of ML/ANNs in terms of their brittleness and failure to generalize.

The presented example utilizes a cell-level ABM, though the approach is potentially modifiable to any complex multi-scale mechanistic model. Cell-level ABMs represent biological systems as interactions between different cell types where each cell type is governed by a defined set of literature-based rules [for a recent review of ABMs in biomedicine see (Sivakumar et al, 2022)]. Such ABMs are desirable for synthetic data generation because: 1) they embody knowledge and spatial interactions not readily replicated with a set of differential equations (which would be readily and misleadingly be reconstituted by training an ANN), 2) they incorporate stochasticity at a generative level (where it exists in biology) and therefore are able to produce the non-Gaussian stochastic distributions seen at the system level and 3) are able to encompass epistemically uncertain or undefined biological features if constructed in a particular fashion that allows the incorporation of epistemic uncertainty in a mathematical object call the Model Rule Matrix (MRM) (Cockrell and An, 2021).

**FIGURE 1:**
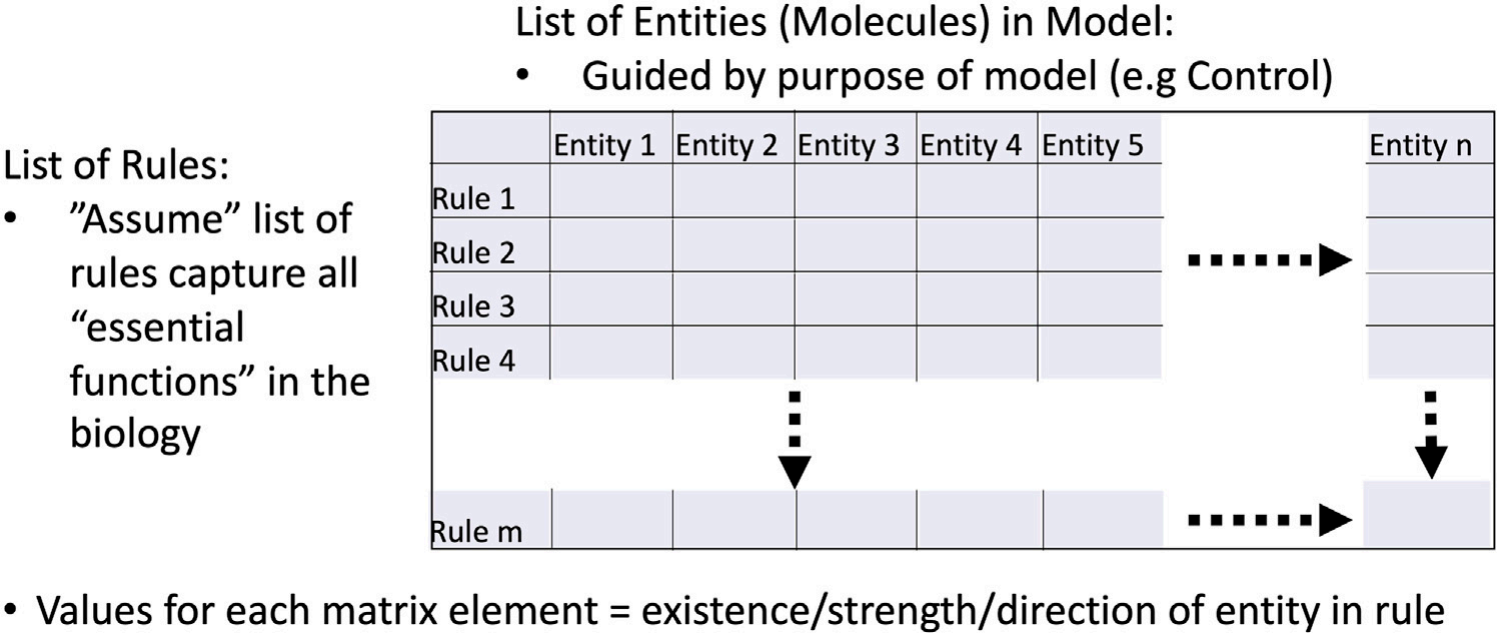
Schematic of Model Rule Matrix (MRM). The MRM is a matrix that depicts the relationship between the entities represented in a mechanism-based computational model (in the columns) and the behavioral rules incorporated in the model (rows). The matrix element values represent the contribution of the particular entity to the cross-referenced rule. The initial MRM for a given model will be relatively sparse, as it will only include the explicitly included rules in the model. However, the initially “0” matrix elements represent the unknown/unrepresented potential connections/contributions of those entities that might be present in the real-world system; it is these matrix elements that will be uncovered as the MRM is evolved in the below presented machine learning calibration pipeline. See below Figures 3, 4 for the result of this process.

The MRM is an interaction matrix between 1) all the entities/molecules/mediators chosen to be included in a simulation model and 2) the rules utilized by the simulation model to represent the biological functions. The numerical values present at each matrix element denote the strength and direction of the contribution of the entity (column) to the functional rule (row). The lack of inclusion of a particular entity in a particular rule is represented by a “0” for the corresponding matrix element. See Figure 1 for a depiction of the components of an MRM. Since every dynamic computational model represents some choice by the modeler of the features and interactions to be represented, the “base” model produces a relatively sparse MRM: this MRM would only include those entities and rules represented in the model and all other matrix elements have values of 0. However, it is readily evident that the necessary selection of what is represented in the model itself represents a *bias* with respect to the full biological complexity (though an unavoidable one) and that there are numerous other potential/inevitable contributions from biological components/molecules/pathways/genes that influence the behavior of the explicitly represented model components. Thus the “0” elements in the base MRM represents a representation of a “latent” space of uncharacterized and unspecified interactions that are nonetheless known to be present in some form at some degree. We assert that it is the gap between the explicitly represented structure of a computational model and the recognized additional potential interactions (by whatever degree of connectivity) that contributes to a model to capture the richness (manifested as behavioral heterogeneity) in the real biological system. It is in this fashion that the MRM utilizes the Maximal Entropy Principle: the potential information content of the model is enriched by the representation of connections that are unknown or electively omitted that become necessary to represent the full heterogeneity of a data set. The enriched MRM is then capable of representing a genetically, epigenetically, and functionally diverse cohort of *in silico* patients able to represent a range of heterogeneous experimental or clinical data.

The process of evolving enriched MRMs involves the application of a ML pipeline that employs both Genetic Algorithms (GAs) (Goldberg and Holland, 1988; Fonseca and Fleming, 1993; Haupt and Haupt, 2004; Cockrell and An, 2018) and Active Learning (AL) (Cohn et al, 1996; Brinker, 2006; Schein and Ungar, 2007; Huang et al, 2010; Tsymbalov et al, 2018). To a simulation model constructed such that the coefficients of the rules in the simulation model can be considered strengths of interactions of their associated variables (and is therefore able to be represented by a MRM) and a data set that manifests a large degree of variability. The ML pipeline identifies the set of MRMs (e.g., set of possible additional connectivity configurations) that are able to encompass the range of variability present in the data set. A more detailed description of the ML pipeline is included in the Supplementary Material and in Refs (Cockrell and An, 2021; Cockrell et al, 2021). The large degree of variability in the data is desirable because the goal is to represent the widest range of biological behavior as possible; having such a target data set expands the descriptive comprehensiveness of an ensemble of MRMs. As opposed to classical parameter fitting, which seeks to find a minimal set (or single) optimal parameter configuration(s), this process does the exact opposite by identifying a very large set of *non-falsifiable* configurations that has the capability of generalizing beyond the training data set and reducing the risk of brittle, over-fitted models. Time series data is particularly desirable, because the requirement of characterizing of behavioral trajectories further constrains the set of non-falsifiable MRMs (because the knowledge-based form of the rules limits their possible behaviors).

**FIGURE 2:**
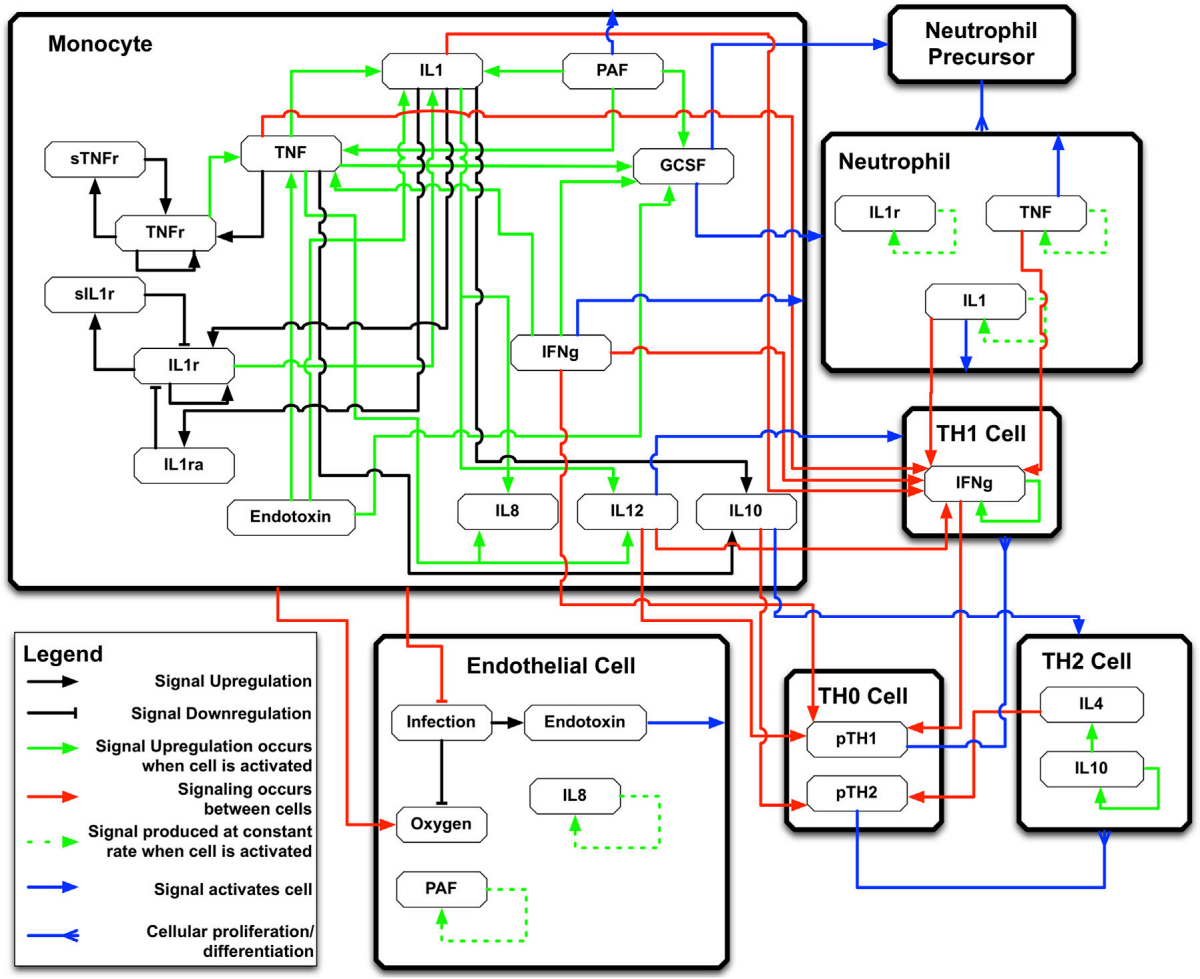
IIRABM schematic diagram. This is a high-level overview of the signaling rules incorporated into the IIRABM. Rules represented include cytokine upregulation/downregulation, cell activation, and cellular differentiation. See Supplementary Material for a more detailed description of the IIRABM. Figure reprinted from Cockrell and An (2018) under the Creative Commons License.

We show an example of this process drawn from Ref (Cockrell and An, 2021), which uses as an example simulation model a previously developed and validated agent-based model (ABM) of systemic inflammation, the Innate Immune Response ABM (IIRABM) (An, 2004; Cockrell and An, 2017). The IIRABM is a two-dimensional abstract representation of the human endothelial-blood interface. The IIRABM simulates multiple cell types, including endothelial cells, macrophages, neutrophils, T-lymphocyte subtypes (TH0, TH1, and TH2 cells) as well as their associated precursor cells. See Figure 2 for a diagrammatic representation of the components and interactions in the IIRABM. Intrinsic biological stochasticity, such as the spatial distribution of cells at initialization or movement direction not governed by chemotaxis and the manifestation of switches governing cellular actions, is represented by the introduction of randomness into the IIRABM; this allows the IIRABM to generate a population distribution of different trajectories from identical parameterizations and initial conditions.

**FIGURE 3:**
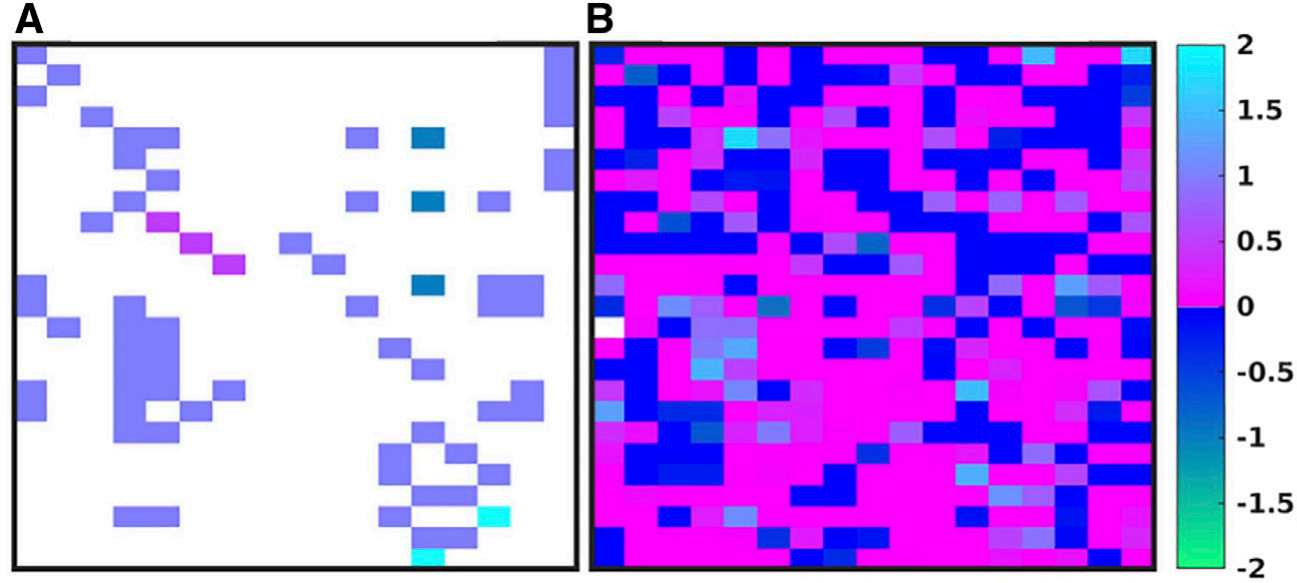
Depictions of the MRM for the IIRABM. There are 17 columns, one for each molecular entity in the IIRABM, and 25 rows, one for each molecular rule in the IIRABM. A heatmap of the original rule matrix is shown in **(A)**, the optimized matrix representative of the valid ensemble is shown in **(B)**. In **(A, B)**, the white blocks represent a matrix element with a value of 0 (e.g., no connection); the dark blue to green represents a negative matrix element; the pink to light blue represents a positive matrix element. The optimization process vastly increases the connectivity of the ABM elements (as would be expected in the true biological system). Figure reproduced from Cockrell and An (2021) under the Creative Commons License.

**FIGURE 4:**
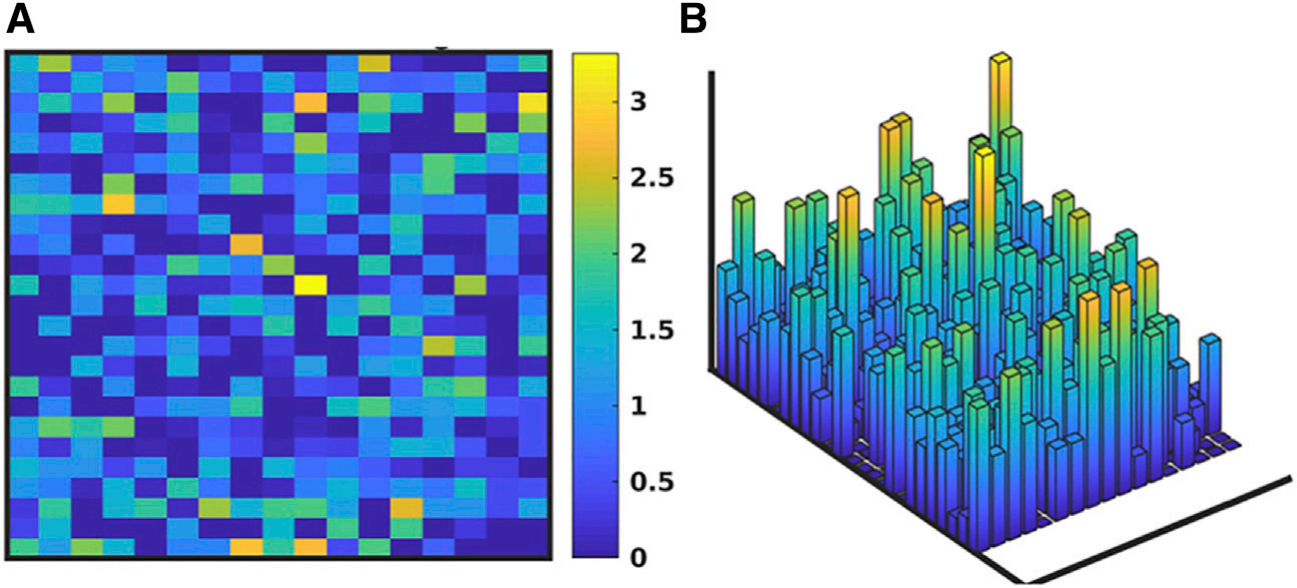
Depiction of ensemble of MRMs. Depiction of the range of values of the MRM for the IIRABM for the valid ensemble of MRMs able to produce data non-falsifiable by the clinical data (consistent with the Maximal Entropy Principle). As with Figure 3, the MRM includes the 17 columns for each molecular entity in the IIRABM and 25 rows for each molecular rule in the IIRABM. **(A)** shows the ranges of theMRM values as a heatmap, where dark blue is a range of 0 and yellow indicates a range of 3.42, with a maximal range of 4.0. **(B)** shows this same data as a 3-dimensional bar graph, where the height of each cell reflects the range of the values for each matrix element. Figure reproduced from Cockrell and An (2021) under the Creative Commons License.

Figure 3 shows the evolution of the IIRABM’s MRM as it is “calibrated” to multiplexed cytokine time series data from a burn population as reported in Ref (Cockrell and An, 2021). The structure of the IIRABM consists of 17 columns representing the biologically relevant molecular entities included in the model rules, and 25 rows each representing a rule in the IIRABM. Figure 3A shows the “base’ MRM as a heat map of matrix elements where the heat map is related to the interaction coefficient present in the rule (we used a heat map representation of MRM values to aid in visualizing the change in the MRM as it is operated on through the GA/AL pipeline). Note that the “base” MRM is relatively sparse, as it only includes the interactions explicitly implemented in the code of the IIRABM. Alternatively Figure 3B (reproduced from Ref (Cockrell and An, 2021) shows an example MRM that has been derived from the GA process of calibrating the IIRABM to encompass the target data set (in this case a publicly available multiplexed mediator data set on burn patients as reported in Ref (Cockrell and An, 2021). What is immediately evident is that the previously sparse “base” MRM has been substantially enriched by the additional of numerous latent “control” interactions, such that the heatmap of the evolved MRM has a similar appearance (in terms of connectivity) as is commonly seen in gene expression/proteomic data. To take this a step further, in order to meet the Maximal Entropy Principle, we do not assume that there is a single “best” MRM that cannot be falsified by the data, and therefore the AL pipeline is used to identify an ensemble of evolved MRMs that show a range of non-falsifiable coefficients as seen in Figure 4 [reproduced from Ref (Cockrell and An, 2021)]. What is evident is that while the number of non-falsifiable configurations is considerable, it is not infinite and does show discernable structure.

The sum effect of this pipeline is a set of simulation model configurations grounded on a known, putative knowledge structure, yet incorporates the sum-total of unknown interactions in that knowledge structure that cannot be falsified by the reference data set. This step fulfills the goal of the Maximal Entropy Principle by minimizing the bias in the resulting functional forms of the simulation model such that when this set of simulation model configurations are run to generate SMTs it provides the widest possible range of possible plausible data to mitigate the limitations of the AI ANN as it trains on it.

## 6 An example of generating SMTs using the ML-MRM approach

**FIGURE 5:**
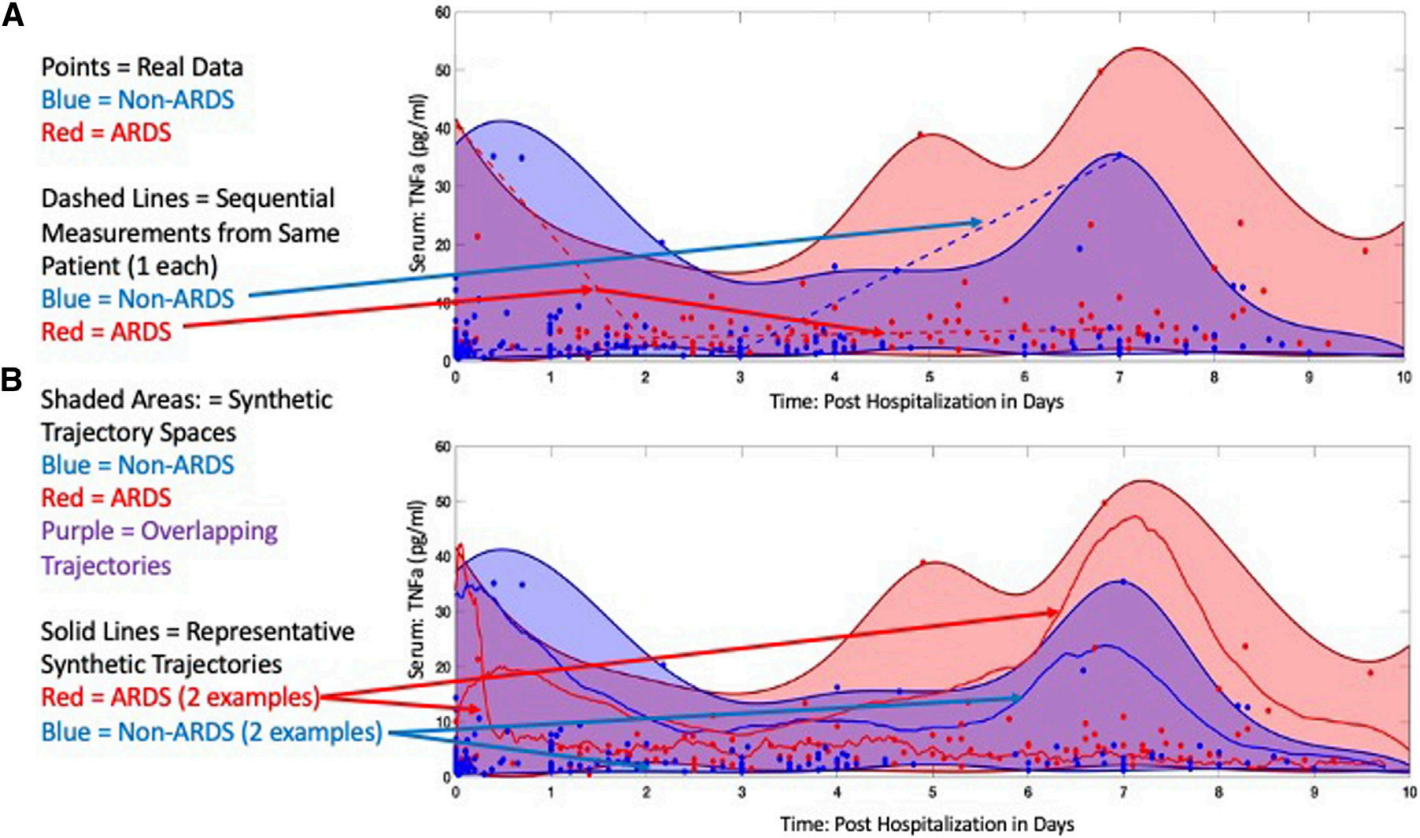
Comparison of experimental and simulated protein concentration trajectories. In **(A)**, we show both simulated and clinically collected blood-serum cytokine measurements for TNFα. Distinct points are the clinically collected data, with red points representing patients that developed ARDS sometime between days 7 and blue points representing patients that did not. The dotted lines indicate representative clinical trajectories seen in the data; note specifically the sparsity of time points for an individual. The shading indicates the boundaries of the model trajectory space for the parameterizations that generate ARDS (red) or not (blue). Note significant overlap between the two spaces given the overlap of data points. However, the key point is that differential parameterizations are able to identify clear regions that are unique to each group. In **(B)**, the three solid lines (two red lines eventually developing ARDS and the blue line not) show actual simulated trajectories of TNFα blood-serum concentrations; note here the continuous nature of the trajectories, which would more accurately reflect the actual underlying biological behavior. Reconfigured from Cockrell et al (2022) under the Creative Commons License.

To demonstrate how SMTs can be generated with this approach, we use the IIRABM within the GA/AL pipeline to create synthetic cytokine trajectories that expand upon data from a cohort of trauma patients to distinguish between those that develop acute respiratory distress syndrome (ARDS) from those that do not. This work is reported in Ref (Cockrell et al., 2022). The IIRABM was calibrated to a clinical data set from The Uniform Services University/Walter Reed National Medical Military Center of 199 trauma patients, 92 of which developed ARDS at some point during the course of their hospitalization, matched with 107 controls that did not develop ARDS. Data elements that were used to calibrate the model include two primary elements: 1) vital signs/laboratory observables necessary to determine SOFA score; for the respiratory compartment, this consists of the partial pressure of oxygen, complete information regarding respiratory support, and blood oxygen saturation, representing aggregate organ function; and 2) time-series blood-serum cytokine profiles consisting of Interleukin-1-beta (IL-1b), Interleukin-1 receptor antagonist (IL-1ra), Interleukin-6 (IL-6), Interleukin-4 (IL-4), Interleukin-8 (IL-8), Interleukin-10 (IL-10), Granulocyte Colony Stimulating Factor (GCSF), Interferon-gamma (IFNg), and Tumor Necrosis Factor-alpha (TNFa), sampled periodically for the duration of the patient’s hospitalization. Simulations of the IIRABM were used to identify parameterizations (which represent varied individuals responding to varied insults) that could not be falsified by the available data, and therefore represented the most expansive potential interpretation of the data given the structure of the IIRABM and its corresponding MRM. Using the nested GA parameter discovery and AL parameterization boundary identification method referred to above [and described in more detail in the Supplementary Material and in Ref (Cockrell and An, 2021; Cockrell et al, 2021; Cockrell et al, 2022)] we identified an ensemble of IIRABM MRMs that were used to perform simulations of burn injury and generated SMTs for those patients that would develop ARDS and those that would not. Representative SMT spaces can be seen for TNFa (as a representative cytokine) in Figure 5 (reconfigured from Ref (Cockrell et al, 2022). Note that several features are present in these plots that reflect what we have noted as important characteristics for SMTs to be used to train ANNs.

1. Note that the raw data (seen as discrete blue or red points) are highly variable, highly overlap, and have variable and shifting distributions over the course of the time series. This pattern is nearly ubiquitous in time series mediator data and precludes the ability to make any informed decision on the statistical distribution of such data (which would be necessary for either a statistical generation of synthetic data, or to inform a non-traditional noise function in a stochastic differential equation). This calls back to the issue of persistent data sparsity, inability to *a priori* assume a statistical distribution and non-discrimination between mediator measurements despite different clinical phenotypes.
2. While there is considerable overlap in the trajectory spaces for non-ARDS (blue) and ARDS (red), there are distinct points at which these spaces separate. But these points of separation are not necessary present at a real-world sampling point, and only become evident in the synthetic data. This demonstrates that real world data is not only sparse in terms of total number of sample sources (Curse of Dimensionality) but also that logistical issues preclude the ability to sample with enough frequency to capture potentially critical bifurcation points.
3. The solid blue and red lines in Panel B show examples of specific simulated trajectories that make up the trajectory space (also note the difference between these projected trajectories and the clinical sequential time series represented by the dotted lines in Panel A). Note that these trajectories are affected both by their parameterized MRM and the stochastic processes included in the IIRABM, but it is exactly these trajectories that are being sought by the AI ANN (if the purpose of the AI requires the ANN to know how the system works). This is a key point that separates this approach to generating synthetic time series data from synthetic/virtual populations, where aggregate outcomes are the target output, as is the case for epidemiological investigations (45–47), or approaches that create distinct virtual populations by fitting the mean mediator values (4–7).

The computational resources presented in the preceding section can be found at: https://github.com/An-Cockrell/IIRABM_MRM_GA

## 7 The importance of useful failure: iterative refinement

Given the known effects of data drift (Nelson, 2015; Baier et al., 2019; Ackerman et al, 2020) and underspecification (D’Amour, 2020) on the degradation of AI/ML system performance in real-world applications, for mission-critical applications (such as many potential biomedical applications), it is important to mitigate the consequences of performance degradation by anticipating, as much as possible when performance falls below some identified threshold. As such, the ability to determine the regimes of applicability of a given model is essential. A primary rationale for the use of synthetic data is preempt the performance degradation by increasing the training set to increase the robustness of the trained AI system (mitigating data drift), but this presupposes that the means of generating the synthetic data does not merely accentuate any bias present in the data used to generate the synthetic data. If a data-centric approach is used to generate synthetic data to train ANNs, the identification of bias or insufficient representation in generating the training synthetic data will not become evident until the ANN fails in its intended use. This is because there is no step that allows for a “reality check” of the synthetic data for molecular time series because no one knows what it is “supposed” to look like, for the reasons of perpetual data sparsity and lack of means of predefining the shape of the data distribution. This is distinct from use-cases where such a reality check can be performed, e.g. image analysis or natural language processing, where the synthetically generated data can be examined for believability. Conversely, SMT generated by cell-behavior mechanistic multi-scale simulation models can provide an intermediate step in the applicability of the generated SMT can be evaluated using the criteria of non-falsifiability to determine if data drift of the synthetic data set is occurring. The ability to perform simulation experiments allow the mechanistic models to be refined through both the process of evolution of an MRM-like parameter space characterization, or modification of the underlying simulation model rules based on new biological knowledge. This iterative refinement process allows for “useful failure,” since the simulation models are transparent with respect to their composition and can be interrogated to determine where their insufficiencies may be.

## 8 Conclusion

In this paper we pose that there is a specific class of data, namely multidimensional molecular-mediator time series data (abbreviated herein as Synthetic Mediator Trajectories or SMT), that presents challenges to traditional means of generating synthetic data to be used for training ANN/AI systems. The specific properties of this data that preclude traditional statistical/GAN methods are:

1. Perpetually sparse data, such that the Curse of Dimensionality cannot be overcome.
2. The variability/“noise” present in this data cannot be assumed to follow an established distribution pattern (e.g., failure to meet the requirements of the Central Limit Theorem preclude an assumption of a normal distribution).

We further assert that while mechanism-based simulations are needed to overcome the above restrictions, there are specific properties that are to be met to overcome the limits of traditional simulation means of generating synthetic data:

1. The mechanism-based simulation models need to incorporate a means of dealing with the perpetual epistemic uncertainty regarding knowledge of cellular-molecular mechanisms (e.g., no *ab initio* physics-based modeling).
2. The means of utilizing these simulation models to generate SMT must maximize the generalizability of the generated synthetic data set, and this means maximizing the expansiveness of the applied parameter space, motivated by the Maximal Entropy Principle.
3. As part of maximizing the expansiveness of the SMT, this means that the simulation method must represent the space of individual trajectories that cannot be falsified by the data. This is because the ultimate goal of the developed/trained ANN/AI is to forecast individual trajectories and/or identify specific controls for individual trajectories.

We intend the focus of this paper to be very specific: to examine a very specific type of biomedical data, namely trajectories of molecular entities measured over a disease course. This is because there are many other types of biomedical data, as we note in the opening sentences of Section 3, that do not require what we propose. However, the use of serial measurements of molecular entities during a disease course is a central method used in biomedical research, most notably in the discovery of cellular/molecular mechanisms, characterization of pathophysiological dynamics and in the development of new therapeutic agents. The generation and use of increasingly granular molecular information about how biological systems work is the primary paradigm in experimental biology, and, in an applied sense, underpins the entire drug development pipeline. Therefore, while this paper is focused on a particular type of data, it does happen to be a type of data that is present across a wide range of biomedical research. Given the importance of such data and the reasonable interest in applying cutting edge ML and AI methods to these problems, it is crucial to develop a means to generate bioplausible SMT to train these systems. The key descriptor here is “bioplausible,” as we recognize that method will not necessarily be a representation of the *fundamental truth*. As noted above, biomedical research operates in a space of epistemic uncertainty and incompleteness, with every hypothesis subjected to the possibility (and perhaps inevitability) of falsification. Rather, the operational strategy in being able to use biomedical knowledge involves being able to find levels of representation that can be demonstrated to be useful. In this sense, the relationship between our proposed methods and reality is analogous to the case of Newtonian Mechanics. The classical understanding of mechanics is broadly applicable to everyday life and has significant predictive power when in its applicable regime (i.e., not quantum or relativistic); however, Newtonian Mechanics alone cannot explain much of the observed motion of our universe. Similarly, while biomedical models suitable to generate SMT have a broad range of explanatory potential, there will certainly be regimes in which the model that generates that data is invalid as these models are, and will be for the foreseeable future, informed by experimental data and not first principles (hence the importance of iterative refinement and “useful failure.”).

Thus, it can be concluded from our argument that while the acquisition of more data is certainly necessary, it is not sufficient to meet tasks that require knowledge of the mechanistic processes that cross causal hierarchies (e.g., cell/molecule to individual). It also provides guidelines as to what mechanism-based simulations must account for to be suitable for ANN training. We have presented a specific example that utilizes a cell-based ABM within a ML-augmented pipeline that maximizes the expansiveness of a SMT, but we make no claim as to the uniqueness of this approach, either in terms of the type of simulation method applied, nor the specific components of our parameter-space characterizing pipeline. We also note that at this time there is no proof that the creation of SMT in this fashion will enhance the training, performance and generalizability of ANN/AIs. Rather, this paper is intended to provide a theoretical basis for why “classical” means of generating synthetic data for AI training (i.e. statistical or physics-based simulation methods) are not applicable for this specific task of generating synthetic multidimensional molecular/mediator time series data, while also providing theoretical basis for why producing SMT using the presented approach “should” work. Our group is actively working on producing a proof-of-concept demonstration that would lend credence to the presented approach, but in the meantime we hope that by presenting the underlying theory and rationale for generating this type of data we can provoke other investigators to work in this area.

## Data Availability

The original contributions presented in the study are included in the article/Supplementary Material, further inquiries can be directed to the corresponding author.
