# Supplementary material for: Generating synthetic multidimensional molecular time series data for machine learning: considerations
Source: Front Syst Biol. 2023 Jul 25;3:1188009. doi: 10.3389/fsysb.2023.1188009 (PMC12342025; doi:10.3389/fsysb.2023.1188009)
Supplement: Supplementary file 1 [file DataSheet1.PDF]

Supplementary Material for "Generating synthetic multi-dimensional molecular-mediator trajectories: Considerations"

This document includes additional detail on the Model Rule Matrix (MRM), the Nested Active Learning Pipeline and the simulation model, the Innate Immune Response Agent-based Model (IIRABM), used in the example presented in the Main Text. The content of this supplementary file is drawn from and slightly adapted from Main Text Reference 46 (which describes the MRM) [1] and Reference 60 (which describes the Nested Active Learning process) [2].

Background on the Innate Immune Response Agent-based Model

The Innate Immune Response ABM (IIRABM) [3, 4] is a two-dimensional abstract representation of the human endothelial-blood interface. Though the IIRABM has been calibrated to simulate blunt trauma and infectious insult, it is an abstract and generalizable model of human response to injury. The IIRABM abstraction models the endothelial-blood interface for a traumatic (in the medical sense) injury and does so by representing this interface as the unwrapped internal vascular surface of a 2D projection of the terminus for a branch of the arterial vascular network. The closed circulatory surface can be represented as a torus, and this two-dimensional surface defines the interaction space simulated by the model. The spatial geometry of the circulatory system and associated organ interfaces are not directly mapped using this scheme. This abstraction reproduces the circulatory topology accessible by the innate immune system and presents a unified means of representing interaction between leukocytes and endothelial surfaces across multiple tissue and organ types. The IIRABM utilizes this abstraction to simulate the human inflammatory signaling network response to injury; see Figure 2 in the Main Text for a diagrammatic representation of the components and interactions in the IIRABM.

The IIRABM simulates multiple cell types, including endothelial cells, macrophages, neutrophils, T-lymphocyte subtypes (TH0, TH1, and TH2 cells) as well as their associated precursor cells. Intrinsic biological stochasticity, such as the spatial distribution of cells at initialization or movement direction not governed by chemotaxis and the manifestation of switches governing cellular actions, is represented by the introduction of randomness into the IIRABM; this allows the IIRABM to generate a population distribution of different trajectories from identical parameterizations and initial conditions. The simulated system dies when total damage (defined as aggregate endothelial cell damage) exceeds 80%; this threshold represents the ability of current medical technologies to keep patients alive (i.e., through mechanical organ support) in conditions that previously would have been lethal.

The IIRABM characterizes the human innate immune response through the simulated generation of a suite of biomarkers, including the pro-inflammatory and anti-inflammatory cytokines represented in the IIRABM. At each time step, the IIRABM outputs the total amount of cytokine present for all mediators in the model across the entire simulation. The ordered set of these cytokine values creates a high-dimensional trajectory through cytokine space that lasts throughout the duration of the simulation (until the *in silico* patient heals completely or dies). We note that stochastic effects can play a significant role in simulation dynamics; even with identical injuries and initial conditions system trajectories diverge such that a statistical distribution of outcome can be identified for each set of initial conditions. The fact that the initial conditions are exactly identical also means that it is indeed stochasticity, not chaos, that leads to the diverging trajectories. While a detailed discussion of this is beyond the scope of the current paper, interested readers can look to Ref [4].

While the IIRABM successfully simulates the human immune response to injury at a high, overall system level (outcome proportions, time to outcome, etc.), it may not always replicate specific cytokine time series. A cytokine time series data set is not a single sequence of numerical values; rather, it is a sequence of ranges, indicating significant heterogeneity clinical response to severe injury or infection across a population, within which the cytokine measurements for a given patient from the cohort will fall. This heterogeneity is challenging because the magnitude of these ranges is not temporally constant. We pose that for a computational model to be biologically realistic, it must be able to generate any physiological state (represented by the measurements/data available) which can be produced by the biology that is being simulated and do so with the appropriate frequency.

We have previously characterized the shapes of the probabilistic "clouds" of multi-dimensional state space of the IIRABM [4]; these distributions, which are more akin to the range of variable behavior generated by biological systems, are too complex to be represented by a small/simple set of stochastic differential equations with an analytically defined "noise" term. This prompts the need to execute the ABM at large scale to effectively capture and more fully represent the population dynamics structure present in a clinical data set.

The IIRABM was the primary simulation model used to develop the concept of the Model Rule Matrix (MRM), and the subsequent design of the machine learning pipeline used to operate on the Model Rule Matrix (machine learning-MRM or ML-MRM pipeline).

Model Rule Matrix (MRM) Introduction:

The MRM was developed to address the gulf between what a modeler chooses to represent in a mechanism-based simulation model and the fact that there will always be some non-representation of known factors and inability to represent an inevitable set of unknown, yet to be characterized elements. This is the state of perpetual epistemic incompleteness discussed in the main text, and for which the Maximal Entropy Principle is intended to guide how this issue is addressed. The MRM is a mathematical object intended to present what is and is not represented in a model in a way that can be operated on regarding a particular data set. While the MRM was developed with agent-based models (ABMs) in mind, it is applicable to any reasonably complex dynamic model as long as that model's "parameters" can provide the role described below. Note that this MRM concept of "parameter" expands upon the notion of parameters as rate constants for kinetic equations. Rather, MRM parameters represent relational values between model component interactions (in terms of strengths and direction of interactions) and the underlying connectivity of the model components. The following text is drawn from and adapted from Ref [1]

Given a multi-scale mechanism-based simulation model, the rules and a set of coefficients that quantify the effect of the rules are stored in a MRM. The rows of the MRM represent the specific component (e.g. molecules/mediators) interaction rules implemented in the simulation model; each computationally relevant component in the model is then represented by the matrix columns. As a simple example, the system of model rule equations for a single cell:

$$M1_{t+1} = M1_t + M2_t$$
$$M2_{t+1} = -M1_t + M3_t$$

are represented by the matrix:

$$\begin{bmatrix} 1 & 1 & 0 \\ -1 & 0 & 1 \end{bmatrix}$$

Where the first column holds the rule coefficients for Mediator 1 (M1), the second column holds the rule coefficients for Mediator 2 (M2), and the third column holds the rule coefficients for Mediator 3 (M3). We note that this is a simplified rule for the purpose of illustration. The matrix is readily decomposable into a one-dimensional vector, which can be operated on using a genetic algorithm. The number of rows in the matrix then is equal to the number of rules represented in the simulation model, and the number of columns is equal to the number of components that could potentially contribute to the effect made by their associated rule. Note that if a particular interaction between model components is not represented then the corresponding position within the MRM contains a "0". Therefore, the MRM presents a compact mathematical representation of the interaction rules present in an ABM, and the initial "base" version of the ABM would have a sparsely populated MRM (e.g. many "0"s) because only non-0 values are those interaction rules explicitly specified in the model. Note that when we describe the "parameters" of the MRM it is a non-traditional usage of the term "parameter:" not only are these value coefficients that determine the strength of a component's contribution to the represented rule, but they also denote the existence of a potential interaction where one had not been explicitly specified in the creation of the initial model.

Introductory Description of Machine Learning- MRM (ML-MRM) Pipeline

The MRM is operated on using a process pipeline that employs different machine learning (ML) methods; we term this the ML-MRM pipeline. In short, the ML-MRM pipeline uses iterated simulation experiments to explore and define a set of MRMs for a given model that cannot be falsified by a given data set. A key point here is that the ML-MRM pipeline inverts the traditional concept of parameter fitting: rather than attempting to find a "best" set of parameters that can fit a particular data set, the ML-MRM aims to find the maximally permissive set of MRM parameters that cannot be invalidated by a particular data set. This inversion of the goal of parameterization is inspired by the Maximal Entropy Principle, which seeks to limit bias in the generative explanation of a data set (as would be seen by restricting such an explanation to only the initial connectivity of a simulation model, and its associated parameter space). The specific relevance to the use-cased of generating synthetic multiplexed molecular/mediator time series data for use in training ANN/AI systems is that the goal of maximal expressiveness drawn from the Maximal Entropy Principle is directly intended to overcome the primary limitation of ANN/AI systems, which is lack of robustness and generalizability due to data drift.

The Genetic Algorithm component of the ML-MRM pipeline functions as follow, and are drawn from Reference [1]. The GA [5-8] is a population-based metaheuristic optimization algorithm that is inspired by biological evolution. The goal of the GA in the ML-MRM pipeline is to evolve a MRM such that the solution replicates not only what is seen in an experimental or clinical data set, but also its associated variance seen in the data by encompassing all the data points present in the data set. The form of the fitness function for the GA is:

$$F = \sum_{i,t} k_i D_{i,t},$$

where

$$D_{i,t} = \left|\max\left(d_{exp,i,t}\right) - \max\left(d_{comp,i,t}\right)\right| + \left|\min\left(d_{exp,i,t}\right) - \min\left(d_{comp,i,t}\right)\right|$$

and where $\max(d_{exp,i,t})$ represents the maximum value (from all subjects at that time point) for the experimental measurement of data element i at time t; $\max(d_{comp,i,t})$ represents the maximum value of computational model output (from all stochastic replicate simulations) for data element i at time t; and the constants k_i allow for varying weights to be placed on individual data elements (i.e. to account for the fact that the raw difference in a protein concentration would not be on the same scale as differential cell populations over time). By summing the differences between the maximum and minimum values of the simulation model output and experimental/clinical data set, this instantiation of the GA seeks to find MRM configurations that generate a range of data which most closely matches the range of data experimentally. It does so by operating on the model parameterization such that, when instantiated in the simulation model, lead it to minimize the above fitness function. We note we are employing GA is a non-standard fashion, where rather than seeking a specific optimal parameterization of the model, we are using the process of convergence of the GA to identify an ensemble of valid (non-falsifiable) parameterizations of the MRM that are able to encompass the variance and heterogeneity of a data set in concordance with the Maximal Entropy Principle. To construct this ensemble of MRMs, we add an ensemble retainment criterion to the GA procedure. The rational for this step is as follows: we recognize that any putative parameterization which generates cytokine trajectories that always lie within the cilnically observed range cannot be invalidated by the data, and are therefore biologically plausible. As the goal of the fitness function is to obtain maximum coverage over the clinical data range (as per the Maximal Entropy Principle), these parameterizations should be retained for inclusion into the final ensemble, as some of these viable parameterizations may otherwise be lost as the population evolves.

During the development of the ML-MRM pipeline, we realized that, in fact, there would be multiple tiers/hierarchies of MRM elements. The near-comprehensive exploration of this MRM-space would be computationally intractable, given the combinatorial complexity of these MRMs. Therefore, we developed a nested ML process that uses a search/optimization method, Active Learning (AL), to more intelligently and tractably characterize MRM-space. In the ML-MRM pipeline, AL is used to determine the boundaries of the GA-derived candidate MRM parameterizations. The description of the AL component of the pipeline is described in Reference [2], with the following text drawn from that paper:

AL is a sub-field of ML that focuses on finding the optimal selection of training data to be used to train an ANN or statistical model [9], and can be used for classification [10, 11] or regression [12, 13]. AL is well suited (in terms of computational efficiency) for model exploration problems where there is a large degree of combinatorial complexity. AL provides a means of "intelligently" searching for boundaries in multi-dimensional parameter space between those parameter combinations that provide a desired output and those that do not. The problem of combinatorial complexity in the selection of model parameters is well-established in the computational/biological modeling communities [14-18], and is inherent in structure of the MRM. The output of the AL workflow is an ANN classifier, for which the classes are biologically plausible (cannot be invalidated by the data) or not. After the application of the GA procedure, there is an ensemble of biologically plausible MRM parameterizations. Each of these parameterizations represents a single point in a high-dimensional space. *AL allows the determination of biologically plausible parameter ranges around those single points in a computationally efficient manner*. The AL procedure begins by defining initial search boundaries, discretization of the search space lying within those boundaries, and creating a set of putative MRM parameterizations with unknown classification. Each MRM parameterization contained within the discretized search space will

then be fed into the trained ANN. and have its associated class predicted. We then posit the existence of some function,

$$y = f(\vec{x}), x \in \chi \subset \mathbb{R}^n, y \in \mathbb{R},$$

which accepts as input a model parameterization and predicts the associated class, and that this function can be approximated given input data from the training set:

$$D_{train} = \{x_j^t, f(x_j^t)\},$$

for $j = 1, \ldots, n$, where x_j^t represents a single parameterization used to train the ANN. The ANN model uses a binary cross-entropy [19] loss function, in which the loss is given by:

$$L = -\sum_{i=1}^{2} y_i \log(\hat{y}_i),$$

where y_i is the ground truth value and $\hat{y}_i$ is the ANN-approximated score. To generate the initial training/seed set, some number of putative MRM parameterizations (approximately 100) are selected from the discretized set. The simulation then uses these putative MRM parameterizations to run a fixed number of stochastic replicates of the input points to determine class membership as defined by the ability of at least one of the stochastic replicates being able to encompass the data points in the data set. This information is then used to train the AL ANN. The algorithm then ranks the remaining unlabeled parameterizations by class-membership uncertainty:

$$\{x_{i+1}\} = \min_x \left(0.5 - P_i(y|x)\right).$$

Those parameterizations whose class (biologically plausible or not) is most uncertain in the current AL ANN are then selected for labeling and the process repeats until a stopping criterion is reached.

At the end of this process there is a set of MRMs (MRM ensemble) that cannot be falsified by a given data set, and subsequent use of the simulation model would operate over this ensemble of MRMs, be it for the generation of synthetic multiplexed molecular/mediator time series data for ML training (as in the current paper) or for multi-modal adaptive control discovery, as we have described previously [20, 21].

References for Supplementary Material

1. Cockrell, C. and G. An, *Utilizing the heterogeneity of clinical data for model refinement and rule discovery through the application of genetic algorithms to calibrate a high-dimensional agent-based model of systemic inflammation.* Frontiers in physiology, 2021. **12**: p. 662845.
2. Cockrell, C., et al., *Nested active learning for efficient model contextualization and parameterization: pathway to generating simulated populations using multi-scale computational models.* Simulation, 2021. **97**(4): p. 287-296.
3. An, G., *In silico experiments of existing and hypothetical cytokine-directed clinical trials using agent-based modeling.* Critical care medicine, 2004. **32**(10): p. 2050-2060.

4. Cockrell, C. and G. An, *Sepsis reconsidered: Identifying novel metrics for behavioral landscape characterization with a high-performance computing implementation of an agent-based model.* Journal of theoretical biology, 2017. **430**: p. 157-168.
5. Fonseca, C.M. and P.J. Fleming. *Genetic Algorithms for Multiobjective Optimization: FormulationDiscussion and Generalization*. in *Icga*. 1993. Citeseer.
6. Goldberg, D.E. and J.H. Holland, *Genetic algorithms and machine learning.* Machine learning, 1988. **3**(2): p. 95-99.
7. Haupt, R.L. and S.E. Haupt, *Practical genetic algorithms*. 2004: John Wiley & Sons.
8. Cockrell, R.C. and G. An, *Examining the controllability of sepsis using genetic algorithms on an agent-based model of systemic inflammation.* PLoS computational biology, 2018. **14**(2): p. e1005876.
9. Cohn, D.A., Z. Ghahramani, and M.I. Jordan, *Active learning with statistical models.* Journal of Artificial Intelligence Research, 1996. **4**: p. 129-145.
10. Brinker, K., *On active learning in multi-label classification*, in *From Data and Information Analysis to Knowledge Engineering*. 2006, Springer. p. 206-213.
11. Huang, S.-J., R. Jin, and Z.-H. Zhou. *Active learning by querying informative and representative examples*. in *Advances in neural information processing systems*. 2010.
12. Schein, A.I. and L.H. Ungar, *Active learning for logistic regression: an evaluation.* Machine Learning, 2007. **68**(3): p. 235-265.
13. Tsymbalov, E., M. Panov, and A. Shapeev. *Dropout-Based Active Learning for Regression*. in *International Conference on Analysis of Images, Social Networks and Texts*. 2018. Springer.
14. Karp, R.M., *On the computational complexity of combinatorial problems.* Networks, 1975. **5**(1): p. 45-68.
15. Sneddon, M.W., J.R. Faeder, and T. Emonet, *Efficient modeling, simulation and coarse-graining of biological complexity with NFsim.* Nat Methods, 2011. **8**(2): p. 177-83.
16. Hopfield, J.J. and D.W. Tank, *"Neural" computation of decisions in optimization problems.* Biol Cybern, 1985. **52**(3): p. 141-52.
17. Edwards, R. and L. Glass, *Combinatorial explosion in model gene networks.* Chaos, 2000. **10**(3): p. 691-704.
18. Neumann, F. and C. Witt, *Combinatorial optimization and computational complexity*, in *Bioinspired Computation in Combinatorial Optimization*. 2010, Springer. p. 9-19.
19. De Boer, P.-T., et al., *A tutorial on the cross-entropy method.* Annals of operations research, 2005. **134**(1): p. 19-67.
20. Petersen, B.K., et al., *Deep reinforcement learning and simulation as a path toward precision medicine.* Journal of Computational Biology, 2019. **26**(6): p. 597-604.
21. Larie, D., G. An, and C. Cockrell, *Preparing for the next COVID: Deep Reinforcement Learning trained Artificial Intelligence discovery of multi-modal immunomodulatory control of systemic inflammation in the absence of effective anti-microbials.* bioRxiv, 2022.
